# Supervisors’ pedagogical role at a clinical education ward – an ethnographic study

**DOI:** 10.1186/s12912-015-0106-6

**Published:** 2015-11-05

**Authors:** Katri Manninen, Elisabet Welin Henriksson, Max Scheja, Charlotte Silén

**Affiliations:** Department of Infectious Diseases, Karolinska University Hospital, Stockholm, Sweden; Karolinska Institutet, Department of Neurobiology, Care Sciences and Society, Stockholm, Sweden; Faculty of Social Science, Department of Education, Stockholm University, Stockholm, Sweden; Karolinska Institutet, Department of Learning, Informatics, Management and Ethics, Stockholm, Sweden

**Keywords:** Clinical education ward, Pedagogical challenge, Supervisory team, Balancing patient-care and student learning

## Abstract

**Background:**

Clinical practice is essential for health care students. The supervisor’s role and how supervision should be organized are challenging issues for educators and clinicians. Clinical education wards have been established to meet these challenges and they are units with a pedagogical framework facilitating students’ training in real clinical settings. Supervisors support students to link together theoretical and practical knowledge and skills. From students’ perspectives, clinical education wards have shown potential to enhance students’ learning. Thus there is a need for deeper understanding of supervisors’ pedagogical role in this context.

We explored supervisors’ approaches to students’ learning at a clinical education ward where students are encouraged to independently take care of patients.

**Method:**

An ethnographic approach was used to study encounters between patients, students and supervisors. The setting was a clinical education ward for nursing students at a university hospital. Ten observations with ten patients, 11 students and five supervisors were included in the study. After each observation, individual follow-up interviews with all participants and a group interview with supervisors were conducted. Data were analysed using an ethnographic approach.

**Results:**

Supervisors’ pedagogical role has to do with balancing patient care and student learning. The students were given independence, which created pedagogical challenges for the supervisors. They handled these challenges by collaborating as a supervisory team and taking different acts of supervision such as allowing students their independence, being there for students and by applying patient-centredness.

**Conclusion:**

The supervisors’ pedagogical role was perceived as to facilitate students’ learning as a team. Supervisors were both patient- and student-centred by making a nursing care plan for the patients and a learning plan for the students. The plans were guided by clinical and pedagogical guidelines, individually adjusted and followed up.

## Background

Clinical practice in various health care settings is an essential part of health care students’ education. These learning environments are meant to support students’ achievement of the learning outcomes and their professional development [1]. Hence, supervision in clinical practice is complex and the role of supervisors as well as how the supervision should be organized are challenging issues for both health care educators and clinicians [1–3]. Clinical education wards are an attempt to create supportive learning environments for students. They are units with a pedagogical framework focusing on training students in real clinical settings with the support from supervisors [4, 5]. While previous research [6–9] has focused on students’ perspectives on learning at clinical education wards, the present study investigates the supervisors’ perspective.

In the present study supervisors are responsible for the pedagogical activities such as guidance and support in students in their learning process and assessment of the learning outcomes. In the literature this role can also be referred as preceptor or mentor. A supervisor can be defined as a professional role model supporting a student, individually and/or as a member of a group, to link together theoretical and practical knowledge and skills in clinical settings. Supervision aims to increase students’ responsibility and ability to work independently [1, 10, 11]. Supervisors have different ways of perceiving their role in students’ learning. Brammer [11] found that when supervisors see the students as future peers, the supervision is focused on students’ learning and understanding of nursing and the role of a graduate nurse. When supervisors focus on teaching students to perform nursing interventions and tasks, the focus is on completing the workload and controlling the students. Supervisors use different strategies and techniques to supervise students. Trust is fundamental for supervision and supervisors need to create a feeling of security and trust in relation to students. Supervision can vary from supervision-centred teaching and instructing to student-centred questioning and reasoning [1, 10].

Clinical education is carried out in various clinical settings in hospitals and in primary care. The learning environments are not always ideal as the main focus of these settings is on patient care [12]. Organizational aspects such as busy workload, lack of time, budget issues and shortage of staff are identified as barriers to supervision. Further important barriers are that supervision is not considered as real work. It is often not seen as a priority and there is often a lack of structure and guidelines for supervision [1, 2, 12–14].

Clinical education wards are established in various clinical settings as a collaboration between clinical and academic partners aiming to enhance students’ learning [4, 6, 8, 15]. Accordingly, clinical education wards are one way to meet the challenges described above. These wards provide clinical practice for one profession or inter-professionally and train students on different levels [5, 16, 17]. Previous studies [7–9] have shown that clinical education wards have the potential to enhance clinical practice and students’ learning. However, these studies focused on students’ perspectives. To further improve our understanding of how the clinical education wards can benefit students’ learning, there is a need for deeper understanding of supervisors’ pedagogical role in this context.

The theoretical framework for this study is based on understanding learning as a process where an individual actively constructs and develops knowledge and skills in interaction with other individuals and the environment [18–21]. Supervision is seen as an essential part of the learning process corresponding to a student-centred questioning and reasoning approach [1, 10]. Supervision is meant to support individuals in gaining new understanding and skills [3].

## Aim

To explore supervisors’ approaches to students’ learning at a clinical education ward where students are encouraged to independently take care of patients.

## Methods

### Design

This study forms part of a project that explores students’ learning at a clinical education ward from the perspectives of students, patients and supervisors using qualitative interpretative approaches. The present study has an ethnographic approach focusing on supervisors’ perspectives. The ethnographic approach can be used to explore social interaction in naturalistic settings by collecting data from multiple sources like observations and interviews [22, 23].

### Setting

The setting for this study was a clinical education ward with eight beds at a department of infectious diseases at a university hospital in Sweden. Four nurses and one nurse assistant serve as supervisors assisted by a clinical lecturer and a physician. Fifteen students do their clinical practice simultaneously for 6 weeks. Nursing students both in the beginning and at the end of their education are trained at the ward. Other health care professions, such as physiotherapist, dietician, occupational therapist and counsellor, are also linked to the ward.

The pedagogical framework used at the ward is based on an interpretation of Mezirow’s theory of transformative learning [19]. This means that students take care of their own patients both individually and in pairs and have supervisors who are responsible for both the patient and the student, guaranteeing patient safety. The students plan, perform and follow up the nursing care as independently as possible with support from the supervisors. The supervisors also make the assessment of the students together with the clinical lecturer. The patients are informed about the organization when they are admitted to the ward.

### Participants

All five supervisors who worked at the ward during the autumn of 2012 participated. They were all women, aged 27–45. Three of the supervisors had started at the ward during the actual term; two of them had worked there for more than one term. All of them had previous experience of acting as supervisors.

Ten patients and 11 students (six in their final year and five in their second year) participated in the observations. For more information about the students and patients, see Manninen et al. [24].

### Data collection

Data was collected through participant observations, follow-up interviews and a group interview. Data triangulation was adopted to explore different perspectives on the research question in order to gain richness in data collection. Ten participant observations were performed by the first author (KM). The observer, wearing a nurse uniform, followed one student during a morning shift each time, except for one observation when she followed two students. In the observations, the students encountered and interacted with both patients and supervisors. Extensive field notes that included both observational notes and reflective notes were taken during the observations. The observational notes included description of activities and interaction and the reflective notes included the observer’s questions and thoughts while observing. After each observation, the observer conducted follow-up interviews with students, patients and supervisors separately. The participants were encouraged to talk about what had happened and their feelings about it. The interviews were audio-recorded and lasted between 4 and 20 min. The observer transcribed the field notes immediately after each observation and reflected and discussed them with one member of the research group (CS). A group interview with the supervisors was conducted after the second student group had finished their practice. This audio-recorded interview was conducted by another member (CS) and lasted 65 min. The participants were asked to discuss 1) their experiences as supervisors at the ward, and in other contexts, 2) what they do and how when supervising students, 3) the interaction between students, patients and supervisors and 4) their thoughts about students’ learning.

### Data analysis

In this study, the data was analysed according to ethnographic procedures [22, 23]. The analysis involved an iterative process of description and probing of the relationships and connections between the data from multiple sources. An interpretation of the data results in an understanding beyond the descriptive level and in an ethnographic approach narratives are a common way to present the results. The narratives are created to describe the actions and interactions and to transform the observations and interviews into text [22].

The data analysis involved several interrelated steps and are described as follows:

1. Transcripts from the field notes, the follow-up interviews and the group interview were read through several times by KM and CS.
2. Events including interactions between students and supervisors or supervising acts were marked and discussed with KM and CS.
3. Identified events were sorted into three groups: 1) supervising situations, 2) what supervisors do (based the observations) and 3) what supervisors, students and patients talk about (based on the interviews). Examples of supervising situations were *planning for nursing care*, *administrating medicine* and *performing medical-technical procedures*. Examples of what supervisors do were *asking what-how-why questions*, *observing* and *discussing*. Examples of what participants talk about were *entrusting the student*, *communication*, *nursing care* (supervisors*); feeling of security*, *following up*, *assistance when needed* (students); *high standard, discovering mistakes early* (patients).
4. The three groups were analysed further by looking for the important aspects of students’ learning and what the supervisors’ challenges were. This resulted in three categories describing important aspects of students’ learning and how supervisors handled them. Two categories described the challenges and how supervisors tackled them.
5. These five categories were subjected to interpretation resulting in four themes describing the supervisors’ pedagogical role at a clinical education ward.

Steps 3–5 were performed by KM and discussed and reflected on with CS. Steps 4–5 were discussed with the whole team (KM, CS, EWH, MS) until agreement was reached. The categories and themes are presented in Table 1.

**Table 1:** Overview of categories and themes. Categories present the important aspects for students’ learning, i.e. what supervisors do and talk about, and themes are an interpretation of them expressing the underlying meaning of the categories

Categories	Themes
Students performing by themselves:	Allowing the students their independence
Trust	
Knowing students and learning outcomes	
Advising, discussing	
Following up	
Students’ learning:	Pedagogical challenges
Stepping back/taking over	
Guiding	
Students on different levels	
Supervisors presence:	Being there for the students
Standing behind and beside	
Communication	
Collaboration between supervisors	
Patient participation:	Applying patient-centredness
Supervisors knowing the patients	
Active/passive patient participation	
Patient safety:	
Knowing student’s ability and patient’s needs	
Following routines and guidelines	

### Ethical considerations

The Regional Ethical Review Board at Karolinska Institutet in Stockholm had approved the study. Participants were informed both orally and in writing that the participation was voluntary and would not affect their employment at the ward and that they were able to terminate their participation at any time without any explanations. They were also informed about confidentiality regarding the data. Prior to the observations a written informed consent was obtained. Patients and students were informed similarly.

## Results

**Fig. 1:**
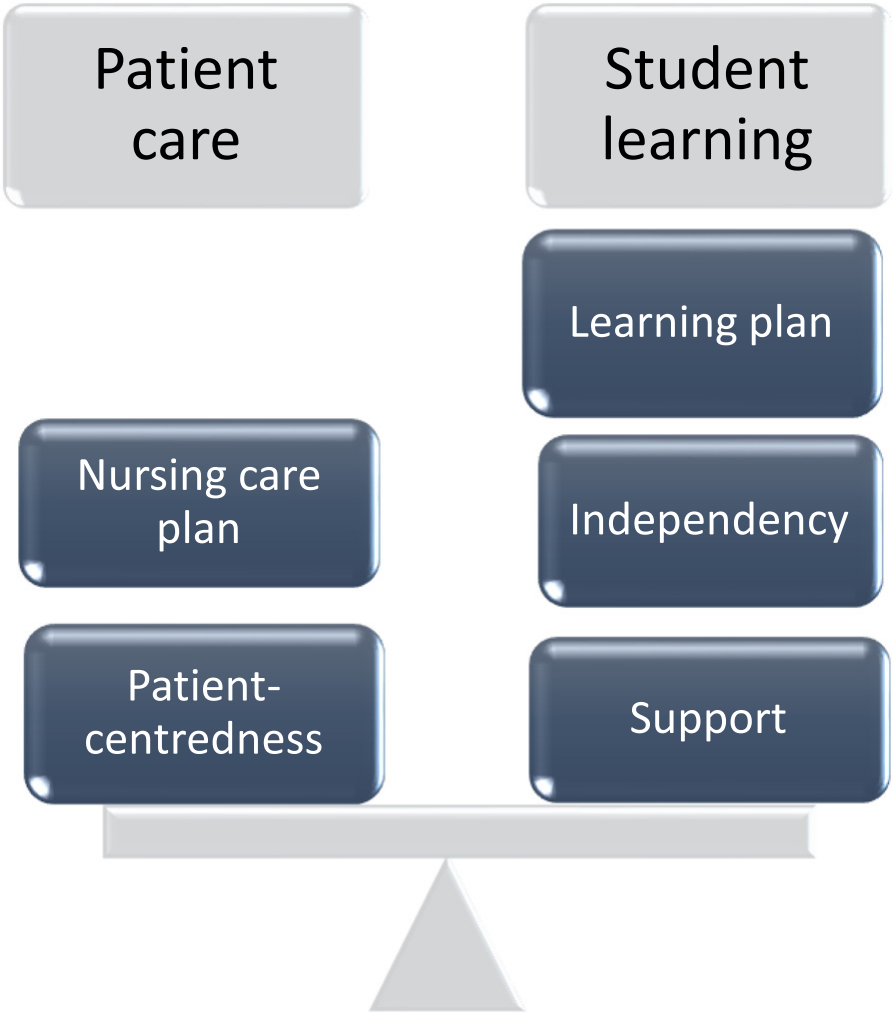
Balancing patient care and student learning. The supervision at a clinical education ward is based on balancing patient care and student learning. The supervisors are focused on the patients, making a nursing care plan for the patients. They are also focused on students, making a learning plan for the students. The plan includes both allowing students to work independently and supporting them when needed

The results are presented as a narrative consisting of the four themes generated from the observations, follow-up interviews and group interview. The narrative describes the supervisors’ experiences of being supervisors in the actual context [22] and it is illustrated with field notes and quotes from interviews. The results show that supervision occurs in all situations and activities at a clinical education ward, some of them indirectly related to patient care, such as organizing the work at the ward. Planning and performing nursing care, medical technical tasks as well as preparing and administrating medicine involve patients directly. The supervisors perform supervision through different acts which are characterized in the themes *allowing the students independence*, *being there for the students* and *applying patient-centredness*. The theme *pedagogical challenge* describes the supervisors’ balancing between patient care and students’ learning. The supervisors’ pedagogical role is illustrated in Fig. 1.

### Allowing the students independence

This theme describes how supervisors allow students their independence. The supervisors are informed about the students’ learning outcomes and their background as individuals and as a group. They trust the students’ ability to perform nursing care and medical technical tasks as well as their capacity to organize the work with support.

Morning meeting at 7 am on Friday. One patient has been admitted during the night shift and the night nurse gives detailed information about the new patient. Then she recounts how the night has been for the other patients. After the report, one of the students leads the discussion about which student will take care of which patient. All students are involved in the discussion. Supervisors also become involved by changing the students’ plans. One of the students has been off sick for a couple of days and the supervisors want this student to take care of one patient by herself, not together with another student.

Observation 1, Field note

The supervisors discuss and reflect with the students continuously, helping them to find answers and solutions to questions and problems related to patient care. Furthermore, the supervisors give students advice and acknowledgement so they feel competent and comfortable enough to perform the care for the patients. The supervisors also follow up and give the students continuous constructive feedback.

The two students had already made their plan and almost performed everything when I checked in with them. They said they needed assistance with the samples, but I was just standing in the room… the student… she did everything by herself. They did the right thing and I felt safe.

Supervisor 1, follow-up interview

You go behind the student so she/he also finds out that they actually already know a lot or that they are learning.

Group interview

### Pedagogical challenges

Supervisors meet different pedagogical challenges connected to students’ learning which re illustrated in this theme. Allowing students independence creates a challenge to balance the patients’ and students’ needs. The supervisors are responsible for patient safety, but at the same time they have to let students act as independently as possible both individually and as a group. Both these responsibilities are equally important for the supervisors. The pedagogical challenge lies in waiting for the students to make decisions without taking over the situations. The supervisors help students reflect on patients’ status, symptoms, nursing interventions as well as the whole situation. Moreover, they follow up on students’ reflections. Another pedagogical challenge lies in stepping back, instead of doing it themselves, when it takes time for the students.

You are so used to thinking that the work is never ending… If you have a minute, you’ll fix something else. When you start to work here [at the ward]… this is the difficult part… to do one thing at a time and let it take time and just stand behind and watch.

Group interview

I noticed that the student was very stressed…I thought that I’d let her work in her pace…I won’t say like hurry up…I’ll wait and see if she says that she can’t manage…but she didn’t…I just let her go on with the work and take it easy.

Supervisor 4, follow-up interview

The supervisors have strategies to reach and maintain the balance between patients’ and students’ needs. The supervisors create a good learning climate by communicating with the students and with each other. They collaborate and work as a team.

We work together… I have not seen everything with every student, but someone else has….

Supervisor 1, follow-up interview

The supervisors follow the clinical guidelines for patient care and the pedagogical guidelines, including the learning outcomes for students. The supervisors are informed about the students’ competencies and based on this information they make a decision as to what kind of support the students need. This support is adjusted according to the individual students’ needs and is based on the learning outcomes. The supervisors follow the clinical guidelines and routines both when they perform tasks themselves and when they instruct the students. They make individual learning plans for the students and held regular meetings to discuss and follow up on pedagogical issues.

Here, the students are in focus…they take care of everything with the patients…I feel that I’m actually permitted to let the students take care of it and we have the procedures for that…so it feels good.

Supervisor 2, follow-up interview

Sometimes you wait until they understand themselves that this is not working out, but sometimes you stop them earlier, you don’t want them to feel that they’ve failed…

Group interview

### Being there for the students

An important aspect in supervision is the presence of supervisors. They can be physically present, close to the students, behind or beside them, and yet letting the students take charge. Sometimes the students are alone with the patients, but the supervisors aim to ensure that the students feel confident in asking for help and support whenever they need it. Additionally, the supervisors and students can collaborate on patient care more equally and at times, the supervisors take over, based on either the patients’ or the students’ needs.

The patient is waiting for surgery, OR will call any time and one blood sample needs to be taken before he leaves the ward. The student tries twice without success and she asks the supervisor to take the sample. The supervisor explains to the patient and to the student that a warm towel for few a minutes might result in that the veins show better. The student fixes the towel and puts it on the patient’s right arm. The patient laughs and says that it feels like being at a spa. The student and supervisor leave the patient for a while to get devices. When they come back, the supervisor asks the student whether she wants to try again. The student says that she prefers that the supervisor takes the blood sample, since they are in a hurry. The supervisor chooses a vein by looking and touching, she explains to the student and to the patient what she is doing. She chooses a vein they took samples from yesterday, she says that it is not an optimal choice, but there are no other options right now. The supervisor succeeds with the blood sample and when the patient sees the blood he says hurray and smiles. The student and the supervisor also smile and they all feel relieved that they managed taking the blood sample before OR called.

Observation 4

Sometimes the supervisors notice that students do not ask for help or communicate with the supervisors. In these cases, the supervisors look for the students, ask questions and offer them help. The supervisors ensure that students get the support they need. They also gather information about the students in several ways: observing and listening to the discussions between the students, talking to patients and other members of the team including the physician. The supervisors are responsive to and make an interpretation and analysis based on the information they get.

### Applying patient-centredness

This theme illustrates the supervisors’ approach to patient care and how the patient-centredness forms the basis of supervision. The supervisors show their concern and responsibility for the patients by working patient-centred through the students but also by making their own plans for patient care. The students are supposed to make their own plan for the patients and the supervisors follow up on the student’s plans, making sure that they do not miss anything.

We discussed the patients’ nutrition and weight loss yesterday, he [the student] wrote it in the nursing plan, and he remembered to bring it up today at the round. I had also noticed that and wrote it down for myself…

Supervisor 5, follow-up interview

Even though the supervisors are focused on the patients, they do not perform all nursing interventions themselves. Instead the students spend time together with the patients, performing the nursing care and other tasks often without supervisors. The supervisors need to be able to let the students to act independently. However, the supervisors need to know when to become involved in situations and sometimes even take over from the students. The supervisors also discuss with the patients to inform themselves about the patients’ experience.

We are responsible for the patients…we meet them together with the students and without them. You talk to the patients and ask how they are doing and how they feel about being at this ward. The patients tell us what has happened and they never complain about the students…they sometimes say it takes time for the students but they don’t complain.

Group interview

## Discussion

The results show that the major task for supervisors when it comes to students’ learning at a clinical education ward is balancing patients’ and students’ needs. As shown in previous studies [1, 2, 12], it is more common that supervision of students is secondary to patient care and the allocation of resources and structures is not always optimal. At a clinical education ward, supervisors regard supervising students and taking care of patients as equally important and consider them as a whole, and not as separate tasks. Consequently, this learning environment allows supervisors to focus on students’ learning processes as well as patient care, which is in line with the findings of Silén et al. [25] regarding physicians as supervisors, as well as Omansky’s [26] and Brammer’s [11] recommendations for organizational management.

Balancing patients’ and students’ needs is a matter of identifying needs, making plans and following them up: a learning plan for students and a nursing care plan for patients. To achieve this, the supervisors emphasize the need to spend time with both students and patients. The supervisors’ approach is characterized by paying attention to students and patients, not focusing in their own actions and thoughts. Being there and focusing on students and patients is in line with Silén’s [27] findings in a study on tutors’ functions in a problem-based learning context. Paying attention to students and their encounters with patients means that supervisors are able to support students on a meta-cognitive level in their learning process. This involves challenging them to reflect on and discuss their thoughts and actions. The supervising acts, such as giving support or taking a step back and reflecting with the students, are based on the students’ needs in actual situations and are subject to change. Supervising on a meta-cognitive level is linked to Mezirow’s [19] theory of transformative learning. This implying that supervisors enhance students’ processing of meaning-making experiences, which results in learning.

The results of this study show further that supervisors perceive their role as facilitating student learning. They do so, as a supervising team, by allowing the students independence, being there for the students and also focusing on patients. Supervisors’ role as facilitators has been emphasized as important by Brammer [11]. Having a team of supervisors is appreciated by the students as it enhances the students’ thinking and reflecting on patient care and on their own learning, as well as further developing their existing knowledge and skills [8, 9, 28]. However, it has also been shown by Manninen et al. [9] that students have concerns about the team of supervisors and that they experience feelings of ambivalence. This means that the students have a wish to be independent but at the same time they feel that they need help and support. Yet they do not communicate this to the supervisors or they wish to have one supervisor who gives them exact instruction about what to do and how to do it. This is something that the team of supervisors needs to handle. One way to help the students to overcome the ambivalence is that the supervisors should communicate explicitly with the students about how they can help and support. It is more common that the supervisors work alone but the results of this study show that working together is beneficial both for the students and for the supervisors. As a team, the supervisors get support from each other by discussing problems and achievements both regarding students and patients. This has also been highlighted by Henderson and Eaton [29]. Working as a team is one way to further develop supervision of students. Each team member is not only aware of the students’ learning outcomes, but also knows the individual students. They are also aware of goals for the individual patients and can tailor students’ learning, taking into account the patients’ needs. The team supports each other as well as students. Another important aspect when facilitating students’ learning is that supervisors have procedures and guidelines to follow both regarding learning and patient care. This is also pointed out by McKown et al. [5]. Based on the results of this study it can be assumed that having a pedagogical framework and following guidelines can be a part of further development of clinical education. Supervision will then not be based on individual supervisors’ own thoughts, but on creating a common understanding and evidence-based knowledge.

A challenge and a need for future clinical practice is to create learning environments where supervision of students and patient care are acknowledged as equally important. In these learning environments supervisors should be given both pedagogical and organizational resources to be able to focus on both patient care and student learning. This study has explored supervisors’ pedagogical role and approaches to students’ learning at a clinical education ward. There is a need for deeper knowledge about supervisors’ pedagogical role and how this role develops over time when working in this kind of context.

### Strengths and limitations of the study

Data triangulation, collecting data from observations and individual and group interviews, was used to enhance trustworthiness [30]. The observations did not include situations with patients who were unable to communicate verbally and this might have affected the results. Furthermore, reflexivity is an important issue in ethnographic research, since the participant observer is actively engaged in observations. In order to enhance reflexivity, reflective notes were taken during the field work and each observation was discussed with another member of the research team. The first author’s pre-understanding of the setting was continuously challenged by the research group. Further, another member of the research team conducted the group interview. The investigator triangulation attempts to increase trustworthiness [22, 30]. In order to extend the applicability of the results to other contexts, the results are related to the theory of transformative learning by Mezirow [19].

## Conclusions

The pedagogical framework at a clinical education ward enables the supervisors to focus on both patient care and student learning simultaneously. Most importantly, they both are considered equally important. Supervisors’ approaches to students’ learning at a clinical education ward is based on finding a balance between taking care of patients and supervising students simultaneously. Finding this balance is a pedagogical challenge which the supervisors handle by being both patient- and student-centred. They make a nursing care plan for the patients and a learning plan for the students. Both plans are guided by the clinical and pedagogical guidelines, individually adjusted and followed up. The supervisors work as a team at the clinical education ward and this collaboration becomes an important part of their pedagogical role to facilitate the student’s learning and independence. Working as a team also turns out to be beneficial for the supervisors in developing their own confidence and understanding of how to support the students.
